# SMS reminders- future in self-care management of diabetes mellitus?

**DOI:** 10.1186/1758-5996-4-31

**Published:** 2012-07-04

**Authors:** Talha Riaz, Haris Riaz, Syed A Hussain, Danish Kherani

**Affiliations:** 1 Department of Medicine, Civil Hospital Karachi, Bab-e-Urdu Road, Karachi, Pakistan; 2 Dow Medical College, Dow University of Health Sciences, Bab-e-Urdu Road, Karachi, Pakistan; 3 Ziauddin University, Karachi, Pakistan

## Abstract

Application of SMS in reminders of medical appointments and delivering medical tests is not new, however its focus on clinical interventions has just begun. Usage of tailored SMS reminders to increase adherence in treatment programs among sick individuals has allowed an interventional role in self-care management of Diabetes Mellitus (DM).

## 

Respected Sir,

Short Message Service (SMS) or Text messaging, is a communication feature among mobile, web or phone communication systems, allowing an exchange of short messages between mobile phones or fixed line devices. Its cost effectiveness and easy accessibility has made it one of the most widely used communication pathways in the world [1]. The integration of SMS in all forms of society, especially lower income populations, has the potential to sideline internet and telephone as the new tool for behavioral intervention in the field of medicine [1].

Application of SMS in reminders of medical appointments and delivering medical tests is not new, however its focus on clinical interventions has only recently begun [2]. Usage of tailored SMS reminders to increase adherence in treatment programs among sick individuals has suggested an interventional role for SMS in self-care management of Diabetes Mellitus (DM) [3].

Self-management of DM, especially type 1 Diabetes Mellitus, is important to avoid acute and long term complications [4].The range of complex requirements of diabetic patients like glucose monitoring, insulin, medication management, psychotherapy, social support and nutrition counseling make self-management of diabetes challenging [5].But use of automated SMS reminders containing diabetic education, cues to action and specific diabetic management suggestions have shown to increase knowledge and improve health monitoring for diabetic patients [6]. Franklin et al established that scheduled, tailored text messaging offered an effected means of supporting adolescents with diabetes and could be adapted for other health-care settings and other chronic diseases [7]. Hussein et al evaluated the feasibility of SMS usage between clinic visits and demonstrated positive effects on glycemic control (via HbA1C) among uncontrolled adult type 2 Diabetes Mellitus (DM) subjects [8]. Although SMS reminders have been particularly useful for teenagers and the elderly [9], Gammon et al documented effective use of SMS as a potential aid for parent–child interaction in self- management of DM in children [10].

Most interventions have shown positive outcomes but the evidence base of SMS based interventions is challenged by methodological limitations and is not yet conclusive. SMS education and cues to action may not be powerful enough to modify many ingrained behaviors. Future studies should use suitable sample sizes to provide greater statistical power for identifying theorized effects and should clearly report the calculations performed to estimate power [3]. Report on proper measures associated with intervention delivery, such as number of sent SMS messages, number of sent SMS replies, how participants treated received SMS messages along with theoretical constructs being targeted in these interventional studies should also be described more elaborately [3]. All these limitations must be addressed in order to enhance further testing and development applicable to this new tool of communication in the self-management of DM.

## **Competing interests**

The authors declare they have no competing interests.

## **Authors’ contributions**

TR thought of the topic. HR and SAH wrote the manuscript. DK and TR reviewed it and made edits. All authors read and approved the final manuscript.

## References

[B1] Katz J Machines that become us: the social context of personal communication technology 2003 Transaction publishers, New Brunswick New Jersey

[B2] Bos A Hoogstraten J Prahl Andersen B Failed appointments in an orthodontic clinic Am J Orthod Dento Orthop 2005 10.1016/j.ajodo.2004.11.014 15775951

[B3] Fjeldsoe BS Marshall AL Miller YD Behavior Change Interventions Delivered by Mobile Telephone Short-Message Service Am J Prev Med 2009 36 2 165 173 10.1016/j.amepre.2008.09.040 19135907

[B4] The DCCT Research Group, authors The effect of intensive treatment of diabetes on the development and progression of long-term complications in insulin-dependent diabetes mellitus. The Diabetes Control and Complications Trial Research Group N Engl J Med 1993 329 14 977 986 8366922 10.1056/NEJM199309303291401

[B5] Kaufman N Internet and information technology use in treatment of diabetes Int J Clin Pract Suppl 2010 166 41 46 20377663 10.1111/j.1742-1241.2009.02277.x

[B6] Boren SA De Leo G Chanetsa FF Donaldson J Krishna S Balas EA Evaluation of a Diabetes Education Call Center intervention Telemed J E Health 2006 12 4 457 465 10.1089/tmj.2006.12.457 16942418

[B7] Franklin VL Waller A Pagliari C Greene SAA randomized controlled trial of Sweet Talk, a text-messaging system to support young people with diabetes Diabet Med 2006 23 12 1332 1338 10.1111/j.1464-5491.2006.01989.x 17116184

[B8] Hussein WI Hasan K Jaradat AA Effectiveness of mobile phone short message service on diabetes mellitus management; the SMS-DM study Diabetes Res Clin Pract 2011 94 1 e24 e26 10.1016/j.diabres.2011.07.025 21840079

[B9] Ferrer-Roca O Cárdenas A Diaz-Cardama A Pulido P Mobile phone text messaging in the management of diabetes J Telemed Telecare 2004 10 5 282 285 10.1258/1357633042026341 15494086

[B10] Gammon D Arsand E Ole Walseth A Andersson N Jenssen M Taylor T Parent–child Interaction Using a Mobile and Wireless System for Blood Glucose Monitoring J Med Internet Res 2005 7 5) e57 16403721 10.2196/jmir.7.5.e57 PMC1550683

